# Orthodontic Metallic Lingual Brackets: The Dark Side of the Moon of Bond Failures?

**DOI:** 10.3390/jfb8030027

**Published:** 2017-07-07

**Authors:** Maria Francesca Sfondrini, Paola Gandini, Andrea Gioiella, Feng Xiao Zhou, Andrea Scribante

**Affiliations:** Unit of Orthodontics and Paediatric Dentistry–Section of Dentistry-Department of Clinical, Surgical, Diagnostic and Paediatric Sciences, University of Pavia, Pavia, Piazzale Golgi 2, 27100 Pavia, Italy; francesca.sfondrini@unipv.it (M.F.S.); paola.gandini@unipv.it (P.G.); andrea.gioiella01@universitadipavia.it (A.G.); fx.zhou@unipv.it (F.X.Z.)

**Keywords:** dentistry, orthodontics, lingual, bracket, base, shear, bond, strength, adhesive remnant index, adhesion

## Abstract

Lingual orthodontics, among both young and adult patients, increased in popularity during last years. The purposes of the present investigation were to evaluate the shear bond strength (SBS) values and Adhesive Remnant Index (ARI) scores of different lingual brackets compared with a vestibular control bracket. One hundred bovine teeth were extracted and embedded in resin blocks. Four different lingual brackets (Idea, Leone; STB, Ormco; TTR, RMO; 2D, Forestadent) and a vestibular control bracket (Victory, 3M) were bonded to the bovine enamel surfaces and subsequently shear tested to failure utilizing a Universal Testing Machine. SBS values were measured. A microscopic evaluation was performed to obtain ARI scores. Statistical analysis was performed at a statistically significant level of *p* < 0.05 to determine significant differences in SBS values and ARI Scores. No statistically significant variations in SBS were reported among the different groups. Conversely, significant differences were shown in ARI scores among the various groups. Clinical relevance of the present study is that orthodontists can expect similar resistance to debonding forces from lingual appliances as with vestibular brackets.

## 1. Introduction

In recent years orthodontists have increasingly faced the problem of patients demanding treatment with more aesthetic appliances. In fact, conventional orthodontic treatment, with vestibular (or labial) visible metallic or ceramic brackets, is often not accepted from adults and subjects with high aesthetic demands. In order to provide solutions to these patients’ requests, lingual (or buccal) orthodontics were introduced [1]. Lingual appliances allow the correction of tooth malposition through fixed therapeutic appliances attached to the lingual tooth surfaces [2]. This technique presents a significantly better aesthetic appearance if compared with conventional vestibular orthodontic treatment; moreover, lingual appliances are practically invisible. 

Recent studies demonstrated that lingual orthodontics could provide treatment outcomes similar to those achieved with labial appliances. However, lingual appliances have been reported to cause additional problems when compared to conventional vestibular appliances such as restriction of mastication. Additionally, speech dysfunctions during pronunciation of some letters, and general oral discomfort have been reported [3,4,5,6,7].

During orthodontic fixed therapy, trauma and application of accidental forces can lead to unwanted bond failures [8]. The most important factor that can reduce bond strength is enamel contamination with water [9,10], saliva [11,12] or blood [13,14], all occurring before bonding. Other factors that can alter bond strength are related to the adhesive system used [15] and bracket base design [16]. Bond failures can influence treatment duration, total costs and chair time [17] which is a serious concern, especially when using lingual appliances [18].

Only few studies [18,19,20,21,22] tested shear bond strength (SBS) to measure adhesion capacity of these brackets. Measurement of residual adhesive after debonding via Adhesive Remnant Index (ARI) scores of lingual [23] appliances is also reported in few papers. Moreover, to our knowledge, no studies compared different lingual brackets versus a control vestibular bracket. 

**Figure 1:**
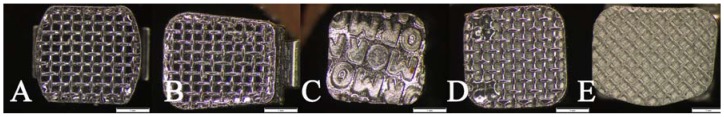
Different bracket bases. Lingual bracket bases: (**A)** Idea (Leone; Sesto Fiorentino, Italy); (**B)** STB (Ormco; Glendora, CA, USA); (**C)** TTR (RMO; Denver, CO, USA ); (**D)** 2D (Forestadent; Pforzheim, Germany); Vestibular bracket base: (**E**) Victory (3M Unitek; Monrovia, CA, USA).

On the basis of these considerations, the present report aims to measure and compare the SBS values and ARI scores of four different lingual brackets tested in comparison with a conventional vestibular bracket (Figure 1). The null hypothesis of the study was that there is no significant differences in SBS values (force recorded to detach the bracket) and in ARI scores (amount of adhesive left on tooth surface after debonding) among the different brackets tested. 

## 2. Results

**Figure 2:**
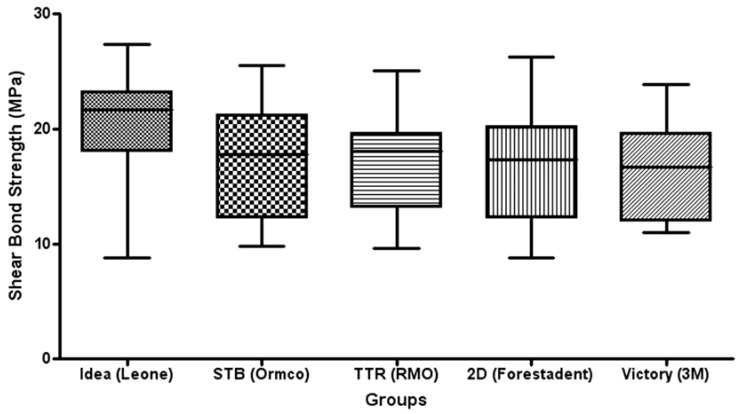
Box plot of shear bond strength (MPa) of the different groups. The band inside the box represents the second quartile. The bottom and top of the box are the first and third quartiles. The ends of the whiskers represent the minimum and maximum. No significant differences were reported among the various groups.

Table 1 shows the results of descriptive statistics. ANOVA detected no significant differences in SBS values among the various groups (*p* > 0.05) (Figure 2). 

Table 2 enlists the ARI scores of the different groups tested. The chi-squared test showed significant differences among various groups (*p* < 0.05). 

A significantly higher frequency of ARI scores of 1 were reported for all the lingual brackets (Groups 1–4), with no significant differences among them (*p* > 0.05). Vestibular brackets showed a significant higher frequency of ARI scores of 0. 

## 3. Discussion

The null hypothesis of the study was partially rejected; no significant differences among the SBS of the various brackets, but differences among the ARI scores were found.

The four different lingual brackets tested (Groups 1–4) showed no significant differences in SBS values among them (Figure 2). During orthodontic treatment, the adhesion between the bracket base and the enamel surface must be sufficient to avoid unwanted bracket debonding due to stresses and masticatory forces. Unwanted bracket detachment can be due to dental trauma, masticatory forces causing fracture and debonding of orthodontic attachments, poor bonding, and low retention of the bracket base. Bracket failure is therefore a common problem in clinical orthodontics that is disturbing for the patients, frustrating for the practitioner, and that increase delays and treatment costs [24,25]. Only a few studies evaluated SBS of lingual brackets. Some evaluated only a single lingual bracket and tested different curing lights [19], the effect of sandblasting [20], and the effect of bracket pretreatment [21]. These studies reported SBS values ranging approximately from 12 to 19 MPa approximately. These data are in agreement with the shear values recorded in the present investigation. Other authors [18] tested four different lingual brackets, evaluating the effect of direct versus indirect bonding technique and influence of sandblasting on bond strength. They reported no significant differences when evaluating bonding technique, whereas a significant effect of sandblasting in enhancing shear strength values was shown. Another study tested SBS of customized lingual bracket bases [22], showing results from 10 to 32 MPa. Higher shear strength values are probably due to customization of bracket mesh, thus allowing higher adhesion forces between enamel and base. On the other hand, in the present investigation, the bases were not customized (as showed in Figure 1). Lingual brackets A (Idea, Leone), B (STB, Ormco), and D (2D, Forestadent) showed the classic mesh structure. Vestibular bracket E (Victory, 3M) showed the same structure but with sandblasted surface. Finally lingual bracket C (TTR, RMO) showed an uncommon bracket surface, with the name of the manufacturer engraved in the base structure.

In the present investigation, no significant differences were reported between lingual brackets (Groups 1–4) and vestibular control bracket (Group 5) in SBS values. To our knowledge, no other study has been reported in the literature having conducted a comparison of different lingual brackets with a vestibular control bracket. 

Some authors stated that a bracket with an adhesion force of 6 to 8 MPa exhibits a SBS sufficient for almost all clinical orthodontic movements [26]; on the other hand, the bonding load should not exceed 50 MPa to avoid enamel damages [27]. In the present work, the SBS recorded with the different appliances were included between these limits. However, minimum and maximum values for SBS forces are still unclear [28].

Moreover, in the present report also ARI scores have been taken into consideration. All the lingual brackets tested (Groups 1–4) showed no significant differences among them, with a higher frequency of ARI = 1. On the other hand, vestibular brackets reported a higher frequency of ARI = 0. No enamel cracks after debonding were reported nor for lingual nor for vestibular brackets. This difference in ARI score distribution is probably due to the different bracket base angulation and design of vestibular bracket if compared with lingual ones. Other studies recorded ARI scores of lingual appliances, showing conflicting results. In contrast with the results of the present report, some studies showed a higher percentage of ARI = 2 and ARI = 3 [18,19]. The difference among the results can be due to various adhesive systems tested in these studies. Conversely, other authors [20] reported results in agreement with our findings, with a higher frequency of ARI = 0. In fact, an ARI score of 0 means that bonding system has a higher adhesion to the bracket and a lower adhesion to the enamel [25]. In this case, clinicians need less time for adhesive removal [29]. On the other hand, an ARI score of 3 means a failure between the adhesive system and the mesh of bracket base [30], thus lowering the risk of damage to enamel structure during debonding procedure [31]. In fact also resin that stay bonded to the tooth and demand removal could imply enamel damage risk, as a high variability of different methods for adhesive removal and tooth polishing has been reported [32]. In the present report, a higher frequency of ARI = 1 has been showed for all the lingual brackets tested, thus showing a mixed adhesion modality. 

As the aesthetic perception of orthodontic treatment needs increases so does the importance of a better understanding of the characteristics of lingual orthodontics [33]; moreover, this knowledge is crucial for clinical treatment success. Similar shear bond strengths and ARI scores between vestibular and lingual brackets should allow lingual treatments with similar debonding rates as those reported with vestibular appliances. In fact, further study is needed in the future, so the results of the present report can be tested more extensively in vitro and clinically confirmed in vivo.

## 4. Materials and Methods

One hundred freshly extracted permanent incisors of bovine mandibular arches were collected from a slaughterhouse. Teeth were stored in a solution of 0.1% (wt/vol) thymol [28]. Tooth selection criteria were decided as follows: no cracks, no caries, no enamel damage. The soft tissues were removed from roots surfaces, and then teeth were embedded in an acrylic resin (Leocryl, Leone, Sesto Fiorentino, Italy). Metallic cylinders (diameter: 15 mm) were filled with resin and the teeth were positioned with the buccal enamel surface exposed with the labial surface parallel to shearing force direction. Lingual brackets were bonded to labial surfaces as bovine vestibular surfaces are smoother and regular, and they are similar to human enamel. Bovine lingual surfaces present rough irregularities, therefore in order to standardize the repeatability of procedures, the vestibular surface has been chosen [25,28].

Teeth were divided using random tables in five groups (20 specimens each) according to the five brackets tested: Group 1 (Idea lingual bracket; Leone), Group 2 (STB lingual bracket; Ormco, Glendora, CA, USA), Group 3 (TTR lingual bracket; RMO, Denver, CO, USA), Group 4 (2D lingual bracket; Forestadent, Pforzheim, Germany), and Group 5 (Victory vestibular bracket; 3M Unitek, Monrovia, CA, USA) (Figure 1).

The labial surface of each specimen was cleaned with pumice (fluoride free) for 10 s with a low speed handpiece and a rubber-polishing cup. The enamel surface was then washed and dried.

After a 30 s acid etching (37% phosphoric acid gel; 3M Unitek, Monrovia, CA, USA), teeth were washed with water and dried with an oil free air stream. Then primer (Transbond XT primer; 3M Unitek) was applied, and the brackets were bonded after a resin (Transbond XT resin; 3M Unitek) application on bracket base. Braces were bonded at the center of the facial surface of the teeth. Excess adhesive was squeezed and removed with a scaler before photoactivated polymerization. A light curing unit (Ortholux XT; 3M Unitek) was used for a total cure time of 20 s (10 s on mesial and 10 s on distal sides of the bracket). After storage in distilled water (temperature: 20 °C) for 24 h, specimens were tested with a universal testing machine (Model 3343; Instron Industrial Products, Grove City, PA, USA). Teeth were blocked with the bracket base parallel to shear force. The bonding surface of the brackets remained perpendicular to the horizontal plane and parallel to the direction of the force to be applied, in an effort to minimize peel and maximize shear during testing. Shear force was applied with a blade connected to load cell [28]. Parameters were set in shear mode. Crosshead was moved with vertical occlusogingival direction at a speed of 1 mm/min [22,25,28].

The maximum of the shearing force needed to debond the bracket from enamel was recorded in newtons and converted into megapascals (ratio of newtons to surface area). After debonding, teeth surfaces and bracket bases were analyzed under 10× magnification using an optical microscope (Stereomicroscope SR; Zeiss, Oberkochen, Germany). In order to record and measure the amount of adhesive remaining on tooth and bracket surfaces, the adhesive remnant index (ARI) score was used [23,25]. This scale allows the allocation of a score (0, 1, 2, or 3) depending on the amount of adhesive detected on the enamel surface (0: no adhesive; 1: less than 50%; 2: more than 50%; and 3: 100% adhesive), thus allowing defining bond failure site. 

The Unit Institutional Committee Board has approved the present study for ethic and feasibility features.

Data were then submitted to statistical analysis with R Software (R version 3.1.3, R Development Core Team, R Foundation for Statistical Computing, Wien, Austria) [34]. For SBS values descriptive statistics were initially calculated (mean, standard deviation, minimum, median, and maximum values for each group). Then inferential statistics were gathered. After data normality assessment (Kolmogorov–Smirnov test) an analysis of variance (ANOVA) was applied. For ARI scores evaluation, a χ^2^ test was performed. A *p* < 0.05 significance level was set for all statistical tests. 

## 5. Conclusions

No significant variations in SBS values were reported among different vestibular and lingual brackets tested. Therefore, the different bracket bases tested showed no difference in terms of adhesion capacity.

Lingual brackets showed a higher frequency of ARI = 1 (less than 50% of the adhesive remaining on tooth surface after debonding). Conversely, vestibular showed a higher percentage of ARI = 0 (No adhesive remaining on tooth surface after debonding). After debonding, no enamel cracks were reported for lingual or vestibular brackets.

## Figures and Tables

**Table 1:** Descriptive statistics of shear bond strength (SBS) of the five groups tested (MPa).

Groups	Mean	SD	Min	Median	Max	Significance
Idea (Leone)	20.42	4.62	8.76	21.68	27.32	
STB (Ormco)	17.12	4.98	9.75	17.77	25.54	
TTR (RMO)	17.19	4.29	9.54	18.07	25.00	n.s. *
2D (Forestadent)	16.74	5.18	8.79	17.30	26.25	
Victory (3M)	16.38	4.28	10.98	16.70	23.89	

* not significant—no significant differences were reported in SBS values among various brackets tested. SD: standard deviation.

**Table 2:** Frequency of distribution of Adhesive Remnant Index scores (ARI; %) with the various brackets tested. ARI Score represents the amount of adhesive that remained on tooth surface after debonding.

Groups	ARI = 0	ARI = 1	ARI = 2	ARI = 3
Idea (Leone)	6 (30%)	10 (50%)	4 (20%)	0 (0%)
STB (Ormco)	1 (5%)	15 (75%)	4 (20%)	0 (0%)
TTR (RMO)	4 (20%)	11 (55%)	5 (25%)	0 (0%)
2D (Forestadent)	9 (45%)	11 (55%)	0 (0%)	0 (0%)
Victory (3M)	11 (55%)	9 (45%)	0 (0%)	0 (0%)

ARI = 0: no adhesive; ARI = 1: less than 50%; ARI = 2: more than 50%; ARI = 3: 100% adhesive [23].
